# Current and Future Molecular Testing in NSCLC, What Can We Expect from New Sequencing Technologies?

**DOI:** 10.3390/jcm7060144

**Published:** 2018-06-09

**Authors:** Simon Garinet, Pierre Laurent-Puig, Hélène Blons, Jean-Baptiste Oudart

**Affiliations:** 1 INSERM UMR-S1147, Paris Sorbonne Cite University, 75270 Paris Cedex 06, France; simon.garinet@aphp.fr (S.G.); pierre.laurent-puig@parisdescartes.fr (P.L.-P.); 2 Department of Biochemistry, Unit of Pharmacogenetics and Molecular Oncology, Georges Pompidou European Hospital, Assistance Publique-Hôpitaux de Paris, 75015 Paris, France; joudart@chu-reims.fr

**Keywords:** lung cancer, molecular analysis, NGS, oncogene drivers

## Abstract

Recent changes in lung cancer care, including new approvals in first line and the introduction of high-throughput molecular technologies in routine testing led us to question ourselves on how deeper molecular testing may be helpful for the optimal use of targeted drugs. In this article, we review recent results in the scope of personalized medicine in lung cancer. We discuss biomarkers that have a therapeutic predictive value in lung cancer with a focus on recent changes and on the clinical value of large scale sequencing strategies. We review the use of second- and third-generation EGFR and ALK inhibitors with a focus on secondary resistance alterations. We discuss anti-BRAF and anti-MEK combo, emerging biomarkers as NRG1 and NTRKs fusions and immunotherapy. Finally, we discuss the different technical issues of comprehensive molecular profiling and show how large screenings might refine the prediction value of individual markers. Based on a review of recent publications (2012–2018), we address promising approaches for the treatment of patients with lung cancers and the technical challenges associated with the identification of new predictive markers.

## 1. Introduction

Lung cancer remains the most common cause of cancer deaths worldwide with more than a million deaths per year [1]. It is divided into non-small cell lung cancer (NSCLC), which accounts for 80–85% of cases, and small cell lung cancer (SCLC). Although tobacco is the major risk factor for lung cancer, 10–15% of patients in Caucasians and up to 40% in Asians are non-smokers. Risk factors and disease etiology remain largely unknown in non-smokers even though hypotheses in Asian populations have been made concerning the role of second hand smoke, cooking fumes, or specific environmental factors. However, interrogation of molecular signatures in Asians led to the conclusion that the elevated rate of NSCLC in Asian non-smokers was not related to second hand smoke [2]. In non-smokers, carcinogenesis is often linked to the presence of somatic molecular alterations in specific oncogenic drivers. The use of selective inhibitors such as anti-EGFR or anti-ALK therapies in patients can lead to tumor shrinkage and prolonged survival. It was rapidly demonstrated that the selection of patients that benefit from targeted treatments could not be based on clinical data. This statement led to the generalization of mutation screening in care settings to identify oncogenic drivers. All patients with stage IV or inoperable lung cancer and all non-smokers with lung cancer independently of cancer type should have tumor molecular testing. Because targetable oncogenic drivers are more common in non-smokers, high-throughput strategies should be proposed in this situation when no frequent alteration is found by basic tests.

Before molecular testing, the first step remains pathological diagnosis and immunohistochemistry (IHC) analysis of protein biomarkers such as thyroid transcription factor-1 (TTF-1), programmed death-ligand 1 (PD-L1), anaplastic lymphoma kinase (ALK) or ROS1. Due to the importance of molecular testing for lung cancer patients, the pathologist must remember to save material for subsequent analysis. If liquid biopsies are validated biomarkers to identify oncogene drivers, molecular tumor testing remains the gold standard at least at diagnosis. Indeed, circulating tumor DNA is contributory in 70% of patients with stage IV tumors.

Comprehensive molecular profiling has revealed major heterogeneity and many different oncogenic drivers have been identified in lung cancer. The implementation of large molecular testing for every patient would ideally inform on all type of alterations, both frequent and rare events. However high-throughput assays are not valuable to all patients in care settings. Whole exome sequencing (WES), RNA sequencing, or large comprehensive tests are not always the appropriate strategy because of tissue type, cellularity, turnaround time, and costs. However, validated druggable alterations need to be accurately identified for all patients, then potential targets might be assessed and finally molecular tumor boards may validate and organize high-throughput tests for patients that need extended screening.

Numerous targeted therapies have been developed in recent years, particularly in lung cancer [3,4,5,6]. Such therapies changed the standard of care for NSCLC stages III and IV from cytotoxic chemotherapies to “specific” first line treatment for selected patients [7]. Initially used in unselected patient populations, most targeted drugs failed [3]. This stressed the need for classifications of tumor subtypes and identification of predictive biomarkers. Initially used in research, next generation sequencing (NGS) revolutionized the approach from single gene sequencing to high-throughput characterization. NGS offers a wide range of possibilities from targeted panels testing a few dozen genes to whole exome or genome sequencing [8]. In addition to its outstanding high-throughput sequencing capacity, NGS has major advantages over first generation sequencing. The quantification of mutation ratio is possible and allows the identification of clonal events in the tumor [9]. Detection cutoffs depend on the coverage depth. For targeted gene panels, the average sensitivity is 2%, enabling the detection of low frequency mutations even when low-inputs of tumor DNA are available. For large targeted panels (more than 300 genes) or exomes, sensitivity is around 10% and it is not recommended to use low quality samples or samples with less than 50% tumor cell content. In research programs, comprehensive pangenomic studies integrate not only mutation testing but transcriptomics data or miRs expression data using RNAseq and miRNA-seq and epigenetics features such as DNA methylation with Methyl-seq, or histone signatures with Chip-seq. These pangenomic studies led by collaborative projects—such as The Cancer Genome Atlas (TCGA) and the International Cancer Genome Consortium (ICGC)—have largely characterized genetic and epigenetic abnormalities of numerous cancer types on large cohorts of patients [10,11,12,13], showing that individual cancer genomes can technically be entirely explored. However, if mutation testing remains feasible, tumor comprehensive analysis translation to routine diagnostic remains a technical and clinical challenge for hospital laboratories.

At present in lung cancer, clinical molecular diagnosis consists of identifying druggable alterations. Clinical molecular biomarkers can be divided into: gene fusions, gene amplifications, and gene mutations. Gene mutations were analyzed since the identification of *EGFR* mutations as predictive markers of response to EGFR tyrosine kinase inhibitors (EGFR-TKIs) and gene fusions or amplifications can be tested at the cellular level by IHC-, or at the cytogenetic level by fluorescent in situ hybridization (FISH). Because larger mutation screenings—including *RAS*, *BRAF*, *MET* (mutations, amplifications), *ERBB2* (mutations, amplifications), *ALK* (mutations) and *ROS* (mutations)—were shown to be useful in the management of lung cancer patients, targeted NGS is progressively replacing single gene testing methods. These focused NGS strategies are easy to handle, low cost, and suitable for FFPE samples and low DNA-inputs. Detection cutoff is low (2%) and allows the identification of mutations and amplifications [14]. More specific NGS focused panels use RNA as input and may identify pre-specified fusions such as ALK, ROS RET, NTRKs, and NRG1.

What can we expect from these technologies that could orient decision-making? Dual testing using a DNA and RNA focus panel should allow also an almost complete detection of known druggable targets and are applicable to most samples and patients. For patients undergoing large-scale cancer molecular studies such as exome, RNAseq, and large targeted panels, genetic counseling should be organized to discuss incidental findings such as mutations in cancer susceptibility genes and informed consent should be obtained.

We will discuss molecular strategies available in routine care for NSCLC screening, we will define strength and weakness and review new biomarkers related to new treatment options, combinations, and treatment sequences.

## 2. 1-Lung Cancer Molecular Screenings, Update on Validated Markers and Emerging Ones

### 2.1. -Mutation Testing

#### 2.1.1. EGFR

The epidermal growth factor receptor (EGFR) was the first oncogenic target to be discovered in NSCLC. The prevalence of *EGFR* mutations ranges from 40% in Asiatic patients [15] to 11–17% in Caucasian patients [16,17]. Smokers or former smokers are associated with a lower incidence of *EGFR* mutation [18]. *EGFR* mutations are mainly associated with female gender and adenocarcinoma histology.

Almost all *EGFR* mutations involve exons 18 to 21. Small in-frame deletions in exon 19 (del 19) represent about 40–50% of *EGFR* mutations [19,20] while p.Leu858Arg amino acid substitution in exon 21 accounts for 30–40% [17,21]. Uncommon *EGFR* mutations, 10–18% of *EGFR* mutated samples are defined as *EGFR* mutations that are neither exon 19 deletions nor p.Leu858Arg substitution [22,23] and include exon 20 insertions and a few exon 18 alterations for the most frequent rare alterations. A heterogeneous group of complex mutations mainly composed of an association of classical mutations and uncommon ones has also been reported [19,20,22,24]. All of these mutations lead to a constitutive activation of EGFR but are not equivalent in terms of EGFR-TKI predictive value [25].

##### First- and Second-Generation EGFR-TKIs

The management of advanced NSCLC has been clearly improved by the development of EGFR tyrosine kinase inhibitors (EGFR-TKIs) during the last decade.

First generation EGFR-TKIs, erlotinib and gefitinib, reversibly bind the ATP-binding site of the EGFR tyrosine kinase domain and inhibit autophosphorylation thereby blocking EGFR-induced activation of the downstream signaling pathways (i.e., Akt–mTOR pathway and mitogen-activated protein kinases (MAPK) pathway) [26]. Numerous clinical trials (IPASS, WJTOG3405, NEJ-002, OPTIMAL, EURTAC, first-signal) have demonstrated an increased progression free survival (PFS) in patients treated by first generation EGFR-TKI compared to platinum-based chemotherapy. These studies mainly enrolled previously untreated patients with common *EGFR* mutation (del 19 or p.Leu858Arg) [27].

Second generation EGFR-TKI, afatinib irreversibly binds the intracellular kinase domain of EGFR, HER2, and HER4 [28]. LUX-lung 3 phase III study showed in previously untreated patients, an improved PFS for afatinib compared to platinum-based chemotherapy (11.1 vs. 6.9 months respectively) [29]. This increased PFS was confirmed in the LUX-lung 6 study [30].

In the LUX-lung 3 and LUX-lung 6 phase III trials, the overall survival (OS) was not significantly longer in the afatinib group compared to the chemotherapy group (23.1 vs. 23.5 months, respectively). However, in *EGFR* del 19 mutated subgroup, OS was significantly higher in the afatinib group in both trials (33.3 vs. 21.1 months in LUX-lung 3 trial and 31.4 vs. 18.4 months in LUX-lung 6 trial, respectively) [31].

The LUX-lung 7 phase IIB clinical trial compared afatinib with gefitinib in the first-line treatment of patients and showed a significant increase in PFS in the afatinib group (median 11 vs. 10.9 months respectively, HR: 0.73, *p* = 0.017) [32]. However, there was no significant difference in OS between afatinib and gefitinib (27.9 vs. 24.5 months, respectively) [33].

All of these results suggest that EGFR-TKIs remain the best first-line therapy in EGFR-mutated advanced NSCLC. The choice of first line between first and second generation is mainly related to different toxicity profiles and to mutation type.

##### EGFR-TKIs Treatment for Patients with Uncommon EGFR Mutated Tumors

Whereas the use of EGFR-TKIs as first-line treatment for patients with *EGFR* mutated tumors is no longer discussed, the efficacy of these treatments in case of uncommon *EGFR* mutations is not clearly defined. Only a few studies have investigated the action of EGFR TKIs on uncommon *EGFR* mutations.

Different studies have evaluated the efficacy of first-generation EGFR-TKIs in the treatment of ‘frequent uncommon’ *EGFR* substitutions p.Gly719X and p.Leu861Gln. These treatments seem to be active on these mutations but remain less effective than in those with common mutations. In 2015, Chiu et al. showed an objective response rate (ORR) and disease control rate (DCR) significantly lower compared with common mutations (ORR: 41.6% vs. 66.5% and DCR: 76.6% vs. 95.1%, respectively) [34]. These results are consistent with those published by Zhang et al. in 2017 [35] and Wu et al. in 2011 [36]. The NEJ002 study showed a shorter OS among patients with uncommon *EGFR* mutations p.Gly719X or p.Leu861Gln compared with common *EGFR* mutations [37]. Despite these results, the National Comprehensive Cancer Network (NCCN) guidelines include exon 18 p.Gly719X and exon 21 p.Leu861Gln as drug-sensitive mutations [38]. Concerning the exon 20 *EGFR* p.Ser768Ile substitution response to first generation EGFR TKIs was lower than that of common mutations [36]. This mutation is not currently classified as a drug-sensitive mutation by NCCN guideline [38].

A post hoc analysis of LUX trials using 32 samples with uncommon *EGFR* mutations and compound alterations (p.Leu861Gln, p.Gly719X, and/or p.Ser768Ile) tested the efficacy of second generation TKI afatinib. Most patients responded to treatment and frontline use of the drug was expanded by the FDA to patients with rare alterations [19,39], suggesting that afatinib might be a good alternative in first line for patients with uncommon sensitive alterations. 

First- and second-generation EGFR TKIs are ineffective treatments on patients with *EGFR* exon 20 insertion mutated tumors [19,31]. Platinum-based chemotherapy remains the best first-line option for these patients.

##### Third-Generation EGFR-TKI

**Figure 1:**
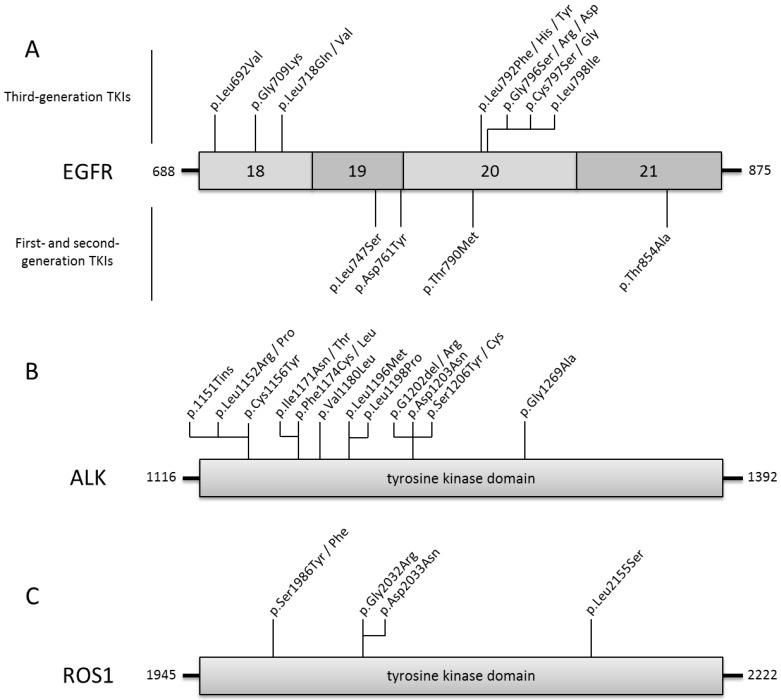
Resistance mutations in EGFR, ALK, and ROS1 drivers. (**A**) Description and gene location of EGFR resistance mutations to first-second and to third EGFR-TKIs; (**B**) description and gene location of ALK Tyrosine kinase resistance mutations to ALK inhibitors described for *ALK* fusions; (**C**) description and gene location of *ROS1* Tyrosine kinase resistance mutations to ROS1 inhibitors described for *ROS1* fusions.

Third-generation EGFR-TKI was developed to specifically overcome the *EGFR* exon 20 p.Thr790Met resistance mutation, which is the most common mechanism of drug resistance to first and second-generation EGFR-TKIs (Figure 1A). Osimertinib (AZD9291) is an irreversible EGFR kinase domain inhibitor targeting the cysteine-797 residue within the ATP binding site [40]. It is effective both against common *EGFR* mutated lung cancers (i.e., deletion in exon 19 or p.Leu858Arg) and exon 20 resistance mutations (p.Thr790Met).

However, Osimertinib remains ineffective against other mechanism of EGFR-TKIs resistance such as *EGFR* exon 20 insertion, *MET* or *ERBB2* amplifications, epithelial-to-mesenchymal transition (EMT) or acquired mutations in *BRAF*, *PIK3CA*, *KRAS*, and *NRAS* genes [41].

The phase I/II AURA clinical trial [42] enrolled patients with advanced lung cancer that progressed after EGFR-TKI treatment. The median PFS was 9.6 months in patients with *EGFR* p.Thr790Met mutated tumors and 2.8 months in *EGFR* p.Thr790Met negative patients. The existence of other acquired resistance mechanisms has not been studied in these patients.

The AURA 3 clinical trial [43] compared osimertinib vs. platinum-based chemotherapy plus pemetrexed in patients with *EGFR* p.Thr790Met mutated tumors who had disease progression after first generation EGFR-TKI. The median PFS was significantly longer with osimertinib compared to chemotherapy (10.1 vs. 4.4 months, respectively) and the ORR was also increased (71% vs. 31%). Osimertinib is now recommended as second line therapy for patients with *EGFR* p.Thr790Met mutated tumors. To confirm the efficacy of osimertinib as a second line treatment, the ASTRIS phase III clinical trial (NCT02474355) is currently in progress and includes patients with advanced or metastatic *EGFR* p.Thr790Met mutation-positive NSCLC that have progressed after treatment with EGFR-TKIs therapy.

##### Resistance to Third-Generation EGFR-TKI

Unfortunately, as described for the other EGFR-TKIs, resistances to osimertinib ultimately develop after a median PFS of 9.6 months [42]. Mechanisms involved in this resistance are not fully understood and appeared to be as heterogeneous as those described for first and second-generation EGFR-TKIs. 

Osimertinib resistance can be divided into EGFR dependent and EGFR independent mechanisms. The first resistance mechanism identified in patients was the tertiary *EGFR* mutation p.Cys797Ser which directly targets the EGFR fixation site of osimertinib [44]. Tumor cells are resistant to all EGFR TKIs when the *EGFR* p.Cys797Ser and p.Thr790Met resistance mutations are located on the same allele, i.e., in cis-position. However, when these mutations are located in trans-position, a combination of first- and third-generation EGFR-TKIs could be administrated [45]. Other resistance *EGFR* mutations (e.g., EGFR p.Leu692Val, p.Glu709Lys, p.Leu718Gln/Val, p.Leu792Phe/Tyr/His, p.Gly796Asp/Ser/Arg, p.Cys797Gly, p.Leu798Ile) [46,47,48] (Figure 1A) and *EGFR* amplification have been described as alternative resistance mechanisms.

EGFR independent resistance mechanisms consist in activation of alternative pathways through different kinds of mutations (e.g., *BRAF* p.Val600Glu, *KRAS* or *NRAS* exon 2–3–4, *PIK3CA* p.Glu545Gln, *AKT*, *PTEN*, or *CTNNB1*) or gene amplifications (mainly *EGFR*, *ERBB2, MET*, *FGFR1*, *KRAS*, *NRAS*, or *PIK3CA*) [49].

Moreover, cellular changes were described as EGFR-TKIs resistance mechanisms. For instance, SCLC transformation was associated with resistance both to first-generation TKI and third-generation EGFR-TKI [50]. Phenotypic alterations, EMT and the acquisition of stem cell features are also consistent mechanisms of resistance to all EGFR-TKIs.

##### Third-Generation EGFR-TKI as First-Line Treatment of EGFR Mutated NSCLC

The FLAURA study [51] is a phase III clinical trial which compared osimertinib to first-generation EGFR-TKI in first line treatment of EGFR mutated NSCLC. This study only included common *EGFR* mutated lung cancers. The median PFS was significantly increased with osimertinib compared to first generation EGFR-TKI (18.9 vs. 10.2 months, respectively) whereas the ORR remained similar (80% vs. 76%).

No clinical trial has compared osimertinib as a first-line treatment versus first-generation EGFR-TKI in the first-line treatment until disease progression followed by osimertinib treatment in second-line for patients with *EGFR* p.Thr790Met mutation-positive NSCLC. Actually less than a half of patients treated with first or second generation EGFR-TKI have access to osimertinib in the second line thanks to the identification of the *EGFR* p.Thr790Met. Osimertinib in the first line has a favorable safety profile and may allow more patients to benefit from treatment. However, up-front resistance and secondary resistance are only partially explored raising the question of second-line treatment in case of acquired resistance. Furthermore, second generation EGFR-TKI was compared to osimertinib.

##### Allosteric Inhibitors of EGFR

Most of EGFR TKIs target the ATP-binding site of the tyrosine kinase domain of EGFR. The low selectivity of these treatments leads to additional toxicities. Moreover, the efficacy of these treatments can be altered by mutations within the ATP binding site (i.e., Thr790 or Cys797). For these reasons, allosteric EGFR inhibitors with different mechanism of action have been developed. For instance, EAI045—that binds an allosteric site outside the ATP-binding site—significantly and selectively modulates kinase activity in EGFR-TKIs resistant mutants. Allosteric EGFR inhibitors targeting the EGFR p.Cys797Ser resistance mutation are considered by some authors as fourth-generation EGFR-TKIs.

#### 2.1.2. BRAF

*BRAF* mutations occur in 2 to 8% of patients with NSCLC [16,52]. The *BRAF* exon 15 p.Val600Glu activating mutation accounts for 50% of all *BRAF* mutations. Other alterations are found in the exons 11 and 15, and are divided into activating (i.e., p.Gly469X, p.Leu597Arg, or p.Lys601Glu) or impaired mutations (i.e., p.Gly466Val, p.Asp594X, p.Gly596Cys) [53]. It results in the activation of the MAPK pathway through an activation of ERK signaling. Impaired mutants have decreased BRAF kinase activity but activate the MAPK pathway through the activation of CRAF signal transduction.

As expected from melanoma data, single BRAF inhibitors (i.e., vemurafenib or dabrafenib) induce cell cycle arrest and apoptosis in p.Val600Glu mutated-NSCLC [53]. Several case reports showed partial or complete response after single-BRAF inhibitor treatment [54], despite short median PFS and OS (5 and 10.8 months, respectively) [55].

The most recent advance in daily clinical practice for metastatic *BRAF* mutated-NSCLC is the association of a BRAF inhibitor dabrafenib with a MEK inhibitor trametinib. In a phase 2 trial, the association of dabrafenib and trametinib was assessed in first line treatment of *BRAF* p.Val600Glu metastatic NSCLC and showed an ORR of 64% [56]. Since June 2017, this combined therapy is now approved by the FDA as first line therapy for patients with *BRAF* p.Val600Glu mutation-positive metastatic NSCLC [57]. Non-p.Val600Glu mutations represents approximately half of all *BRAF* mutated NSCLC. In-vitro study confirmed that several non-p.Val600Glu *BRAF* mutations in exon 11 and 15 could also be sensitive to dabrafenib and trametinib combination [58].

#### 2.1.3. MET

The MET receptor tyrosine kinase is part of aberrant signaling networks in many cancer types, including lung cancer. MET dysregulations mainly involve gene amplifications and *MET* exon 14 splice site mutations (METΔ14) that are markers of response to MET inhibitors. Both are not exclusive. Other type of *MET* mutations, including point mutations involving the MET TK domain, are rare and their value as markers of response to inhibitors needs to be evaluated for each case [59].

METΔ14 alterations are detected in approximately 3–4% of lung adenocarcinomas, and *MET* amplification from 1 to 5% [60]. In patients with pulmonary sarcomatoid carcinomas that are not RAS mutated, METΔ14 is a recurrent [61]. MET is involved in oncogenic signaling, metastasis, and development of secondary resistance—notably to first-generation EGFR-TKIs [62]. Basically, there are two situations were *MET* testing could help treatment decision: patients with a non-KRAS, BRAF, EGFR, or HER2 tumor for which the identification *MET* as a driver could lead to specific treatment and patients with *EGFR* mutated tumors secondary resistance. In these two situations, MET inhibitors have been tested in combination with EGFR TKIs: in a randomized *EGFR* wild type cohort of 111 patients, PFS was significantly improved in the cabozantinib group (4.3 months), erlotinib plus cabozantinib group (4.7 months) compared with erlotinib alone (1.8 months) [63]. Another randomized phase II trial tested the combination of onartuzumab—an antibody binding to the extracellular domain of c-Met- in combination with erlotinib. PFS and OS were improved in the MET-positive population [64]. Responses to crizotinib have been observed in a small study and is under trial on larger cohorts [62]. Met inhibition showed clinical benefit for patients with METΔ14-driven NSCLC and large clinical trials directed toward *MET*Δ14 may validate selected therapy for those patients. METΔ14 testing should then be part of lung cancer testing. The high variability of splicing alterations may render interpretation of unknown variants challenging. Splice prediction algorithms may be of help but in some cases, RNA analysis to identify the METΔ14 mRNA could be necessary to validate the functional impact of the alteration. Some NGS fusion panels integrate MET analysis for that specific purpose [65].

#### 2.1.4. KRAS

*KRAS* activating mutations are found in nearly 30% of samples and is up to now used as an exclusion biomarker. *KRAS* mutated tumors are more frequent in smokers and rarely harbor other druggable drivers. Co-mutations include *PI3KCA* and *STK11* but the use of PI3K or mTOR inhibitors has not led to any recommendations. Patients with *KRAS* mutated tumors do not benefit from targeted therapy. Trials testing the impact of MEK inhibitors have failed to demonstrate any benefit [66]. Drugs that specifically block the most frequent *KRAS* mutation in lung cancer (p.Gly12Cys) are under development. These drugs target the *KRAS* p.Gly12Cys mutation that is linked to tobacco exposure. Finally, immunotherapy may also be a treatment option for patients with *KRAS* mutated tumors. Different results suggest that smoking status may be a predictive marker for survival benefits to immunotherapy, possibly due to the existence of a high mutation load in tumors from smokers.

#### 2.1.5. PI3KCA

It is likely that PI3KCA might become by itself a predictive marker. However, the presence of *PIK3CA*/*AKt*/*mTOR* pathway co-mutation was shown to confer resistance to gefitinib in EGFR mutated NSCLC. Larger series are needed to confirm this finding.

### 2.2. Fusion Testing

#### 2.2.1. ALK

ALK rearrangements are involved in 3–7% of NSCLC. In 2007, the first described fusion-gene was located in the short arm of chromosome 2 as the result of a fusion between echinoderm microtubule-associated protein-like 4 (EML4) and ALK genes [67]. Other fusion partners—such as KLC1, TFG, or KIF5B—were then identified in NSCLC. ALK-rearrangements lead to a constitutively active oncogenic fusion protein which signals through different signaling pathways such as MAPK or JAK-STAT. In addition to gene fusions, ALK point mutations and amplifications have also been described but the link between these alterations and the response to ALK inhibitors is not well documented. 

ALK fusion should be part of lung cancer routine diagnosis for all stage IV patients as it is easy to detect using immunohistochemistry (IHC) as a screening tool. No restriction to a specific group of patients should be done.

Three generations of ALK inhibitors are now available for the treatment of ALK-rearranged NSCLC. In a phase 3 clinical trial comparing first-generation ALK inhibitor, crizotinib vs. chemotherapy in first line treatment in ALK-rearranged NSCLC, median PFS was significantly longer with crizotinib compared with chemotherapy (10.9 vs. 7.0 months, respectively). Moreover, ORR was also increased with crizotinib (74% and 45%, respectively) [68]. Crizotinib is now considered as a standard first line treatment of ALK-rearranged NSCLC.

Second- and third-generation ALK inhibitors were developed to overcome several resistance mutations to first-generation ALK inhibitor. Second-generation ALK inhibitors, ceritinib, and alectinib, are now both approved as a first line treatment of ALK-rearranged NSCLC. The ASCEND-4 phase III clinical trial compared ceritinib vs. platinum-based chemotherapy for first-line therapy of ALK-rearranged NSCLC. The median PFS was significantly increased in the ceritinib group compared with chemotherapy group (16.6 vs. 8.1 months, respectively) [51]. Moreover, ceritinib significantly improves PFS of patients with crizotinib-refractory ALK-rearranged NSCLC [69]. In the same way, the 12-month event-free survival rate was significantly increased with alectinib compared with crizotinib in the first line treatment of ALK-rearranged NSCLC (68.4% vs. 48.7%, respectively). Unlike crizotinib and ceritinib, alectinib is also effective in central nervous system progression [70]. Lorlatinib is the third-generation ALK and ROS1 inhibitor. A phase-3 clinical trial (NCT03052608) is now recruiting patients to compare lorlatinib and crizotinib in the first line treatment of advanced ALK-rearranged NSCLC. 

Recent studies have addressed the impact of ALK fusion variants on response to ALK inhibitors. Indeed, in vitro studies suggested that sensitivity to ALK inhibitors could differ between variants. In vivo, the most frequent variants are V1 and V3. No significant difference was found for OS, PFS, and progression pattern between patients with tumors harboring V1 or V3 fusion transcripts. In patients treated in third line by lorlatinib after first- and second-generation ALK inhibitors, V3 was associated with longer PFS. However, this result needs to be validated in larger series. The main difference between ALK variants is the rate of secondary resistance mutations with more mutations and more p.Gly1202Arg mutation detected in V3 variants. This could impact the choice of second-line TKI treatment.

Finally, the choice of first line treatment should take into account brain metastasis and be determined by balancing efficacy and toxicity as long as there is no clear molecular evidence to select one or the other.

#### 2.2.2. Resistance to ALK-Inhibitors

As described for EGFR-TKIs, almost all patients treated with ALK-inhibitors ultimately relapse on therapy, generally within 12 to 24 months. Mechanisms leading to the resistance to ALK inhibitors are either ALK-dependent or ALK-independent mechanisms. Different ALK tyrosine kinase domain mutations (exons 20 to 29) were described, leading to a constitutive activation of ALK (e.g., Leu1196Met, p.Gly1269Ala, and then p.Gly1202Arg, p.Ser1206Tyr, p.Val1180Leu, p.Cys1156Tyr) (Figure 1B) [67]. ALK resistance mutations were firstly described after treatment with crizotinib, but seem to be more common after treatment with second generation ALK-inhibitors [71]. Second generation ALK-inhibitors overcome some crizotinib resistance mutations (e.g., p.Leu1196Met or p.Gly1269Ala) but fail to show activity against *ALK* p.Gly1202Arg mutated tumors. On the other hand, the ALK-inhibitor resistance may be induced by activation of alternative downstream pathways as amplification of tyrosine kinase receptors genes such as *EGFR, ERBB2*, or *cKIT* [72]. In addition, EMT has also been described as a resistance mechanism to ALK-inhibitors. The identification of secondary resistance mutation should drive sequential therapy of different generations of ALK-inhibitors. Indeed, inhibitor efficiency depends on the presence of resistance mutations. Lorlatinib is the only inhibitor to be efficient in case of p.Gly1202Arg. Finally, the identification of an ALK independent mechanism may point out another druggable driver.

#### 2.2.3. ROS1

ROS1 rearrangements are uncommon fusion genes occurring in 1–2% of NSCLC, approximately half as common as ALK-rearrangements [73]. ROS1 fusion were identified as potential driver mutations in NSCLC, leading to constitutive kinase activity [73]. Patients with ROS1-rearranged and ALK-rearranged tumors share similar clinical profiles: they are significantly younger and more likely to be non-smokers compared to ROS1 negative group, with a higher prevalence in Asians. Metastatic patterns are slightly different between both groups with more brain metastases and extrathoracic metastatic sites for AKL-rearranged tumors [74]. Crizotinib demonstrated its efficiency against ROS1-rearranged patients, in two independent phase II prospective studies, with a concordant ORR of 72% and 70% in respectively two cohorts of 50 and 53 ROS1 positive patients and a median PFS of 19.2 and 15.9 months respectively [4,75]. Tolerance is generally consistent with the safety profile evaluated in ALK-positive patients. ROS1 screening should be tested upfront as crizotinib is now approved for first line treatment. However, FISH is often performed only in the case of negativity of first line tests.

PFS are often longer in patients with ROS1 rearranged tumors as compared to ALK and only a few mutations were described in ROS1 tyrosine kinase domain as mechanism of crizotinib resistance. In addition upregulation of bypass signaling pathways have been reported. ROS1 p.Gly2032Arg and p.Asp2033Asn remain the most frequently observed crizotinib resistance mutations [74]. ROS1 p.Ser1986Phe and p.Ser1986Tyr mutations were also described to confer resistance to crizotinib but remain sensitive to lorlatinib [76] (Figure 1C).

#### 2.2.4. RET

*RET* fusions were identified in a small subset of NSCLC (around 1% of frequency). According to a meta-analysis on 6899 NSCLC, *RET* fusion gene occurs at significantly higher frequencies in young (<60 years old) female, Asian, and nonsmoker patients. These features are shared with other fusion genes. No impact was detected on prognosis and TNM stage of tumor [77]. No specific targeted drug is yet available for RET-rearranged tumors. However, multikinase inhibitors sunitinib and alectinib are approved, with a limited benefit in term of response (16 to 47%) and PFS (two to seven months). Carbozantinib and vandetanib were also tested in clinical trials; PFS and OS were 5 and 10 months, respectively. Recently, a resistance mutation (*RET* p.Ser904Phe) was identified in a CCDC6-RET fusion tumor in a patient that developed secondary resistance to vandetanib suggesting that similar type of resistance mechanisms as for other targeted drugs can occur [78]. Specific drugs are expected soon with better effects [79,80]. Molecular routine screening of RET rearrangement in front line might become mandatory in the future, RET fusions could be better identified along with other hotspot fusions using NGS fusion panels. 

#### 2.2.5. NTRK

*NTRK1* fusions have recently been described as driver in a subpopulation of lung cancers, about 0.1–3% [81,82]. Despite this low frequency, *NTRK* fusions are an interesting target because of initial reports of NRTK-inhibitors showing a dramatic tumor response and suggesting that the selective inhibition of this pathway is a promising therapeutic approach [82,83]. Entrectinib—a multikinase inhibitor- and LOXO-101—a pan-NTRK inihibitor—are currently under clinical evaluation [83]. The existence of targeted therapies makes *NTRK* fusions a promising biomarker that should be investigated thanks to NGS pan-fusion gene panels or IHC.

#### 2.2.6. NRG1

*NRG1* fusions have emerged as uncommon alterations in lung adenocarcinomas and especially in invasive mucinous lung adenocarcinoma (IMA). NRG1 fusions activates the ERBB2/ERBB3 signaling pathway [84]. A durable response with afatinib was first reported in a patient harboring a *NRG1* gene fusion [85]. However, others reported that response was not achieved with afatinib in four NRG1-rearranged patients, while an exceptional response was observed with anti-ERBB3 monoclonal antibody therapy [86]. Those data suggest that ERBB3 inhibition may be more optimal than ERBB2 inhibition, but larger series are required. So far, *NRG1* fusions are not tested in clinical routine, however NGS fusion panels and RNAseq strategies allow *NRG1* fusion detection. In non-smokers with IMA, *NRG1* fusions should be tested as the identification of this driver has a direct clinical impact.

#### 2.2.7. Gene Fusion Detection

One of the most remarkable advances relative to NSCLC personalized medicine is the ability to detect fusion genes with targeted panels using RNA. Until now, *ALK*, *RET*, and *ROS1* rearrangements were analyzed using either IHC or/and FISH methods. However, FISH is time consuming, expensive and difficult to interpret, thus only *ALK* is constantly tested in routine. For rare rearrangements, FISH is secondarily performed for ROS1 or RET rearrangements when mutations are negative. In daily practice, the low quantity of tumor material does not always allow an extensive study of all putative targets, successively. New NGS fusion panels are now available, offering the possibility of studying rearrangements from low RNA inputs. Basically differences rely on the possibility of detecting all fusion partners or a subset of frequent partners and on the number of fusions analyzed [87]. These data suggest that NGS may provide an effective and accurate alternative to FISH testing for the detection of *ALK* and *ROS1* rearrangements in clinical routine, and offers the possibility of large screening of other rare rearrangements with potential clinical value [87,88,89,90]. Fusion panels work on RNA, they have been optimized for low inputs FFPE-RNA, however quality needs to be checked and long-time storage of FFPE samples is not recommended. Some systems allow the use of total nucleic acids (combined DNA and RNA extraction), enabling mutation testing and subsequent fusion testing on a unique sample.

### 2.3. Technical Evolution in Clinical Molecular Testing

#### 2.3.1. From Single Gene to Multi-Gene Testing/Panels

Single gene testing or restricted hotspot testing methods were developed to screen for *EGFR* p.Leu858Arg mutation or deletions within the exon 19. The identification of rare alterations with a validated clinical impact such as rare *EGFR*, *MET*, or *BRAF* variants enlarged testing coverage and led to the implementation of the clinics high-throughput tests. NGS and especially targeted NGS were rapidly validated for sample FFPE samples and implemented in diagnostic laboratories. For lung cancer, there are easy-to-use and affordable commercial panels that differ slightly but cover the important targets—*EGFR*, *KRAS*, *BRAF*, and *MET*. These panels are referred to as targeted NGS panels as they focus on hotspot regions and frequently altered genes, with a direct and known consequence on therapy, diagnosis or prognosis. These panels have been validated by various studies: Shao et al. showed a concordance rate of 100% on 61 tumor samples previously profiled. Lih et al. compared 380 mutations previously identified in cell lines: the assay achieved sensitivities of 100% for 64 single nucleotide variants SNVs, nine SNVs at homopolymer regions, and 11 large indels, 83.33% for six indels, and 93.33% for 15 indels at homopolymer regions. Thus, NGS can now be considered a first line technology [91,92,93,94]. 

NGS time workflow from sample to results is longer than single gene testing and at some point, clinicians might wonder why they should wait for NGS data while *EGFR* testing is necessary to treat patients. If NGS provides a wider analysis, results are available within a week. To shorten delays for hotspot alterations, prescreening with mutant specific probes can be part of the testing pipeline in order to provide a quick answer for first line treatment. Then, NGS data can be included in the molecular report.

What can we expect from NGS data? We recently showed in a large series of lung cancer patients that besides allowing the identification of *EGFR*, *KRAS*, and *BRAF* mutations, NGS identified a potential driver in 36% of patients (*FGFR*, *ERBB2*, *AKT*, *MAP2K1*, *STK11*…) [14,94,95,96]. Numerous experimental drugs are under development [97], and a large molecular characterization could be mandatory in the coming years. 

Many different panels are being developed, including more genes, tumor mutation load (TML), MSI status determination, and fusions. These comprehensive panels will bring answers and questions. Large NGS panels drive more information and more questions when variants of unknown significance VUS, of unknown predictive value, of predictive value in another cancer type are identified in genes that are potential driver [98]. The link between detection and clinics is not always easy, however international databases help to provide information for each variant identified, combining the functional effect on proteins and response to treatment. Methods for high-throughput functional evaluation are being developed and could offer a fast and accurate improvement for data interpretation [99].

**Figure 2:**
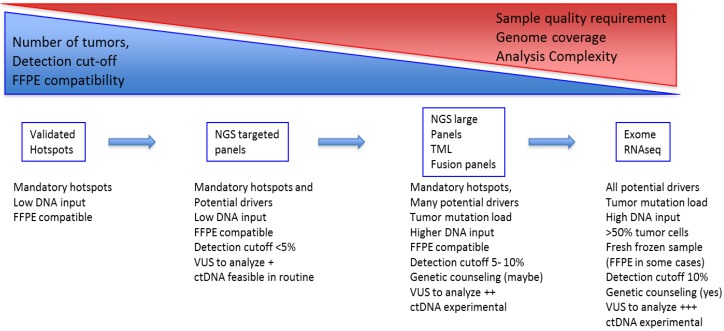
Lung cancer molecular screening options. Figure 2 shows the different technical options developed to identify oncogene drivers in lung cancer from single gene tests to WES including methods’ specificities, mutation cut-off, genomic coverage/panel size, and sample requirements.

The increase in the number of genes in panels raises different problems—technical issues: panel validation, quality assessment and quality control may be tough; clinical issues: the management of VUS, the comprehensive analysis of network of mutations, and the management of incidental findings. Notably, it is now possible to perform a somatic exome in clinical routine. Genetic counseling should be mandatory before somatic exome sequencing in patients with lung cancer so patients may be advised on the possibility of incidental findings and the options for future management and eventually family planning (Figure 2).

#### 2.3.2. From Tissue Testing to Circulating DNA

The emergence of secondary mutations and treatment resistance was seen for all targeted therapies used in lung cancer treatment monitoring as a major challenge for oncologists as evolution of tumor cell genetic profiles and molecular heterogeneity have been linked to resistance. To facilitate molecular monitoring and to limit iterative biopsies, circulating tumor DNA (ctDNA) can be used to identify tumor genetic alterations.

The existence of circulating nucleic acids has been known since 1948 [100], but their potential applications have only been identified in the last few years. Circulating cell free DNAs (ccfDNA) are produced by cell apoptosis, necrosis, or active excretion, and circulate freely in the blood. The recent identification of a fraction originating from the tumor—the circulating tumor DNA (ctDNA)—in patients with malignancies enlarged dramatically the potential use of ccfDNA as a predictive biomarker. Different methods allow the detection of tumor mutation in ccfDNA, and NGS has been adapted to analyze liquid biopsy specimens with good accuracy [101].

Three types of biomarkers can be detected in blood: ctDNA, circulating tumor cells (CTC), and exosomes [102,103]. CtDNA is the most promising of these biomarkers, as the easiest to handle in clinical routine. Various applications of ctDNA are being developed, for diagnostic, prognostic and theranostic purposes. Theranostic value is the most evident application in clinical routine. Numerous studies have been performed to compare ctDNA analysis to match tumor samples: sensitivity is approximately 50–70% and specificity 90–99% [104,105] depending on the studies [106]. The sensitivity is related to the low amount of ctDNA among ccfDNA and to the global amount of ccfDNA that challenges the limit of detection of sequencing technologies. Bioinformatic methods are being developed to discriminate a true mutation at low frequency in ctDNA from background noise [101] and sequencing methods were adapted to improve sensitivity [107]. However, ctDNA cannot be detected in 20–30% of patients. The absence of circulating DNA in some patients might also be clinically meaningful as many studies have shown that no or low ctDNA at diagnosis was related to a better outcome and ctDNA decrease upon treatment is linked to response, PFS and OS.

The second major application is the detection of resistance mutations during targeted treatment: ctDNA can avoid the inherent disadvantages of tissue rebiopsy. When patients progress on first or second line EGFR TKI therapy, the alternative is to look for the *EGFR* p.Thr790Met mutation and switch to a third generation TKI. Liquid biopsy is a good surrogate to re-biopsy and might reflect tumor heterogeneity [108]. It should be proposed as the first line option to monitor EGFR-TKI resistance but re-biopsy is recommend if ctDNA testing is negative [105]. Resistance mutations to ALK-inhibitors can also be detected on ctDNA: in a cohort of 31 patients, McCoach et al. showed that 16 samples (53%) contained 1–3 ALK resistance mutations [109]. ctDNA could, in the future, have wider clinical application as a prognostic marker and a marker of response to treatment independent of treatment type [110]. A recent study on 177 NSCLC highlighted that high ctDNA concentration was and independent prognostic factor for progression-free survival and overall survival. However, concentration changes during treatment did not correlate with radiological CT response [111]. We analyzed prospectively the clinical impact of ctDNA independently of molecular profiles and first line treatment, we found that ctDNA at baseline was an independent marker of poor prognosis, with a median OS of 13.6 versus 21.5 months and a median PFS of 4.9 versus 10.4 months. At first evaluation (E1) after treatment initiation, residual ctDNA was an early predictor of treatment benefit as judged by best radiological response and PFS [112].

#### 2.3.3. Predictive Markers of Response to Immune Checkpoint Inhibitors, Focus on Genetic Determinants

Recent changes in the treatment of patients with advanced lung cancer include the use of immune checkpoint inhibitors (ICIs). Treatment with ICIs can lead to durable responses in some patients but molecular determinants are still being investigated to better select responders. Sensitivity to ICIs is mainly multifactorial, involving tumor genetics background, immune cell infiltrates, and the level of immune-modulators such as PD-L1 or PD1 expression. However, we’ll focus on genetic determinants related to improved survival in lung cancer patients treated with ICIs.

The hypothesis that tumor immune response is activated by antigenic specific peptides and that at least a subgroup of these tumor specific antigens originate from tumor mutations lead to investigating the impact of tumor mutational load on response to ICIs. Indeed, responsiveness to ICIs was first documented in highly mutated cancers such as melanoma and tobacco-related lung tumors [113,114] pointing out that tumor mutation burden, neoantigen load, and response to ICIs were possibly linked.

#### 2.3.4. Driver Mutations as Predictive Markers

Indeed there are evidences that non-smokers with *EGFR* mutated or *ALK* fusion positive tumors do not do well with ICIs. Patients with an identified driver EGFR, ALK or ROS1 should not receive first line ICIs even though tumor cells may express high PD-L1. Upregulation of PD-L1 is not rare in *EGFR* mutated or *ALK* rearranged lung tumors [115] and was related to activation of ERK or mTOR signaling [116]. In second line treatment if the *EGFR* p.Thr790Met mutation is not present patients should be offered chemotherapy [117]. A recent study showed that after EGFR-TKI relapse, ICI treatment was associated with a 2.1 and 1.3 month PFS for *EGFR* Thr790Met-negative and Thr790Met-positive patients [118]. Moreover, ICIs do not improve OS compared to docetaxel in this setting [119]. 

In smokers, *KRAS* and *TP53* co-mutation could be predictive of response to immunotherapy. TP53 was shown to increase expression of immune checkpoints and was linked to interferon-gamma signature. Moreover, *KRAS*/*TP53* mutated samples showed a favorable immune infiltrate and a higher mutation burden [120].

In contrast, *LKB1*/*STK11* mutations in association or not with *KRAS* were related to a lack of response to immunotherapy [121]. This could be related to specific immune environment linked to *LKB1*/*STK11* mutated tumors [122,123].

#### 2.3.5. Tumor Mutational Load (TML) as a Predictive Marker

In lung cancer, somatic mutation load was related to tobacco exposure and to a specific molecular smoking signature. Tobacco induced DNA damage is linked to mutation counts and subsequently to response to ICIs [124]. Recently, an ancillary study of the CheckMate 026 clinical trial explored TML predictive value in a population of lung cancer patients with a PD-L1 expression of 5% or more. The main result from this phase 3 trial was that nivolumab was not associated with significantly longer progression-free survival than chemotherapy. However, TML was assessed in a subgroup of patients using exome sequencing. PFS was longer in the subgroup of patients with high TML defined as >243 mutations per exome or >8 mutations/Mb (median, 9.7 vs. 5.8 months; hazard ratio for disease progression or death, 0.62; 95% CI, 0.38 to 1.00). No difference was observed for OS. It was attributed to treatment crossover. No overlap was found between PD-L1 expression and TML however patients with both PDL1 > 50% and TML high experienced longer PFS.

Based on different studies, high TML seems predictive of response to ICIs, however some patients with low TML respond to treatment and some with high TML have short PFS.

TML is the surrogate marker of tumor neoantigen load (TNagL). Different studies have shown that neoantigen load can be estimated using algorithms that take into account various parameters, including peptide binding to patients’ specific HLA isoforms. TNagL is much lower than TML with only a few neoantigens present even when TML is high [125]. High TML increases the chance that, at random, neoantigens are synthetized by tumor cells. Due to the importance of neoantigens in cancer immunotherapy, TNagL is an attractive biomarker to identify responders to ICIs.

#### 2.3.6. Quantification of Tumor Mutational Burden

Although WES sequencing is actually the gold standard, TML was also investigated using NGS targeted panel. Different strategies have been tested and compared to WES data to validate TML by targeted NGS. Altogether, results showed that good correlations are obtained with WES if TML is determined using large comprehensive panel over 1 Mb. However, mutation cut-offs and a clear definition of low and high TML still need to be validated [126,127]. Finally, the identification of repair pathway defects such as MMR deficiency which is rare considering lung cancer and mutation in DNA polymerases POLE and POLD1 are surrogate markers of TML [126].

## 3. Discussion and Conclusions

**Figure 3:**
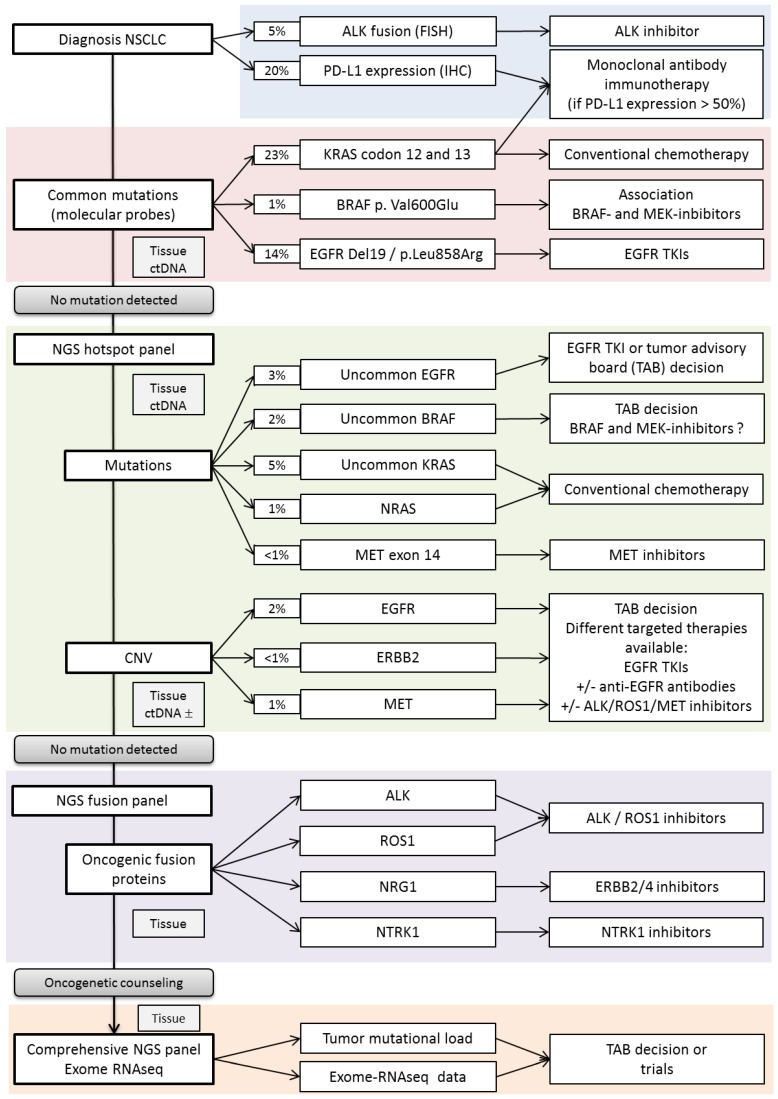
Lung cancer testing algorithm, an example in clinics. Figure 3 shows the different levels of molecular testing from single gene to WES, the expected findings, and potential clinical impacts.

While treatment decisions are determined by cancer stage, molecular alterations drive medical care for patients with advance stage lung tumors. Indeed, targeted therapies have proven to be effective therapeutic approaches and were related to treatment response in selected patients. Many reviews have discussed the clinical value of molecular alterations in lung cancer. However, the access to broad molecular screenings as part of routine care will change the clinical management of lung cancer patients in the near future. Small molecular subgroups of patients are identified with potential drivers and drugs are being developed (BRAF, RET, NTRKs, and NRG1). In parallel, recommendations concerning therapeutic sequences are changing (EGFR), molecular changes in the course of treatment need to be explored to identify secondary resistance alteration and adapt treatment (ALK ROS1) and immunotherapy brings new biomarkers to clinic. Molecular testing is required for all patients with advanced lung cancer to select the optimal first line treatments. Our challenge is to develop comprehensive molecular analyses to optimize treatment choices, combinations, or sequences at diagnosis and during follow-up. An example of lung cancer testing algorithm is summarized in Figure 3. Technological progresses in genomics have made it possible to provide comprehensive molecular tests using small biopsies and FFPE lung cancer tissues. NGS was applied to WES or RNA sequencing many research programs, it is now used as a diagnostic tool in clinical laboratories—but what can we expect from these technologies in care settings? We know sample requirements vary depending on the gene panel size and the type of analyses (DNA or RNA) but basic NGS molecular screenings are feasible in most cases. 

Test performances will vary due to different sensitivities, specificities, sequencing depths, coverages and also due to the samples themselves (age, preservation conditions, tumor cellularity…). Test performance should be mentioned to the clinician and NGS workflows should be validated by external quality control programs. 

Clinicians have to be aware that WES or large panels are not suitable for all samples. Due to lower sensitivity (150X coverage depth), WES may miss mutations in samples with low tumor cell content as compared to targeted panels (>1000X coverage depth) and subclonal populations may be more difficult to identify by exome sequencing.

NGS turnaround time ranges from a week to a few weeks. As a fast turnaround time may be critical to selecting first line treatment, multiplex PCR assays focusing on frequent mutations may still be useful. Indeed, in our experience, concordance between PCR assays and NGS is very good and PCR assays allow identification of *EGFR* and *KRAS* alterations within two days in more than 35% of samples [14].

Clinical interpretation of VUS identified by NGS platforms may be difficult. So the development of molecular tumor boards to discuss treatment options is mandatory for patients with tumors harboring VUS in known drivers and case reports should be collected and stored to educate and inform the community on the clinical impact of rare variants. Moreover, NGS—and especially WES—identifies many alterations in potential drivers, co-drivers, or tumor suppressors. The clinical interpretation of networks of alterations remains a hard task that has no validated clinical value yet. 

Testing strategies must evolve to take into account the increase of new biomarkers, new targeted agents, new combination of drugs, and the necessity to not only diagnose but also monitor treatment responses. One might expect that next generation sequencing technologies will enable selection of the patients most likely to gain from targeted therapy and will ultimately inform clinical decision-making.
